# DL_ANALYSER Notation for Atomic Interactions (DANAI): A Natural Annotation System for Molecular Interactions, Using Ethanoic Acid Liquid as a Test Case

**DOI:** 10.3390/molecules23010036

**Published:** 2017-12-24

**Authors:** Chin W. Yong, Ilian T. Todorov

**Affiliations:** 1 Scientific Computing Department, Science and Technology Facilities Council, Daresbury Laboratory, Sci-Tech Daresbury, Warrington WA4 4AD, UK; ilian.todorov@stfc.ac.uk; 2 Manchester Pharmacy School, Faculty of Medical and Human Sciences, University of Manchester, Manchester M13 9NT, UK

**Keywords:** atomic interactions, hydrogen bonds, hydrophobic interactions, ethanoic acid, molecular dynamics (MD), DL_F Notation, DL_POLY, DL_FIELD, DL_ANALYSER.

## Abstract

The DL_ANALYSER Notation for Atomic Interactions, DANAI, is the notation syntax to describe interactions between molecules. This notation can annotate precisely the detailed atomistic interactions without having to resolve to diagrammatic illustrations, and yet can be interpreted easily by both human users and computational means. By making use of the DL_F Notation, a universal atom typing scheme for molecular simulations, DANAI contains the expression of atomic species in a natural chemical sense. It is implemented within DL_ANALYSER, a general analysis software program for DL_POLY molecular dynamics simulation software. By making references to the molecular dynamics simulations of pure ethanoic acid liquid, it is shown that DL_ANALYSER can identify and distinguish a variety of hydrogen bond and hydrophobic contact networks through the use of the DANAI expression. It was found that the carboxylic groups preferentially orientated in a “head-to-tail” conformation to form hydrogen bonds between the carbonyl oxygen and hydroxyl hydrogen, resulting in a series of linear structures that intertwined with pockets of methyl clusters.

## 1. Introduction

The study of solution chemistry is important to understand all aspects of chemical and structural interactions among the solvent and solute molecules. Such studies are important in industrial gas–liquid dissolution processes [1], crystallisations of organic molecules in the pharmaceutical industry [2] and biologically important processes occurring in lipid bilayers, ion transports and water-mediated protein cavities [3,4]. There are various experimental techniques such as scattering techniques [5,6], which provide local structural data, as well as dynamics and relaxation behaviour of molecular interactions. On the other hand, computational techniques such as molecular dynamics (MD) [7] are particularly suited to tracking both the dynamic and structural evolution of many-body systems such as solutions and liquids at atomistic levels. Indeed, the MD technique was originally developed for simulating the dynamics of simple liquids [8].

Perhaps one of the most important aspects in studying solution chemistry is the interplay between the hydrogen bond (HB) interactions and hydrophobic interactions. For instance, physical properties such as miscibility, solubility and boiling and melting points depend on the nature of dipole strength at the molecular levels, of which strong inter-molecular interactions, such as hydrogen bonds, can result in unusual physicochemical properties of molecular systems.

**Figure 1:**
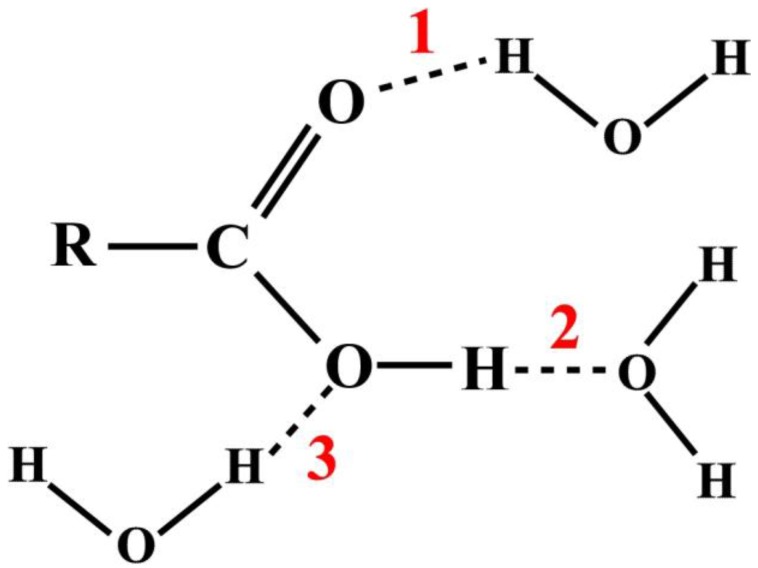
Diagrammatic illustration of hydrogen bond interactions between a carboxylic acid and water molecules; dashed lines represent hydrogen bonds (HB). The numbers 1 to 3 indicate possible sites at the carboxylic group where the HB can form.

In a typical chemistry text book and virtually all research publications, the studies of atomic and molecular interactions are usually illustrated by some textual annotation or arbitrary diagrammatic representations [9,10,11]. For example, consider the HB interactions between a carboxylic acid and water molecules as shown in Figure 1. There are three possible sites, as the numbers indicate, where the hydrogen bonds can form, represented by dash lines.

While such an approach conveys clear pictorial information to the readers, there is no quantitative information to indicate the relative frequencies of the HB formations. On the other hand, MD techniques can provide complete detailed descriptions of the atomistic structures in molecular systems. Such wealth of information is often extracted based on the radial distribution functions [12], that indicate the overall packing of atoms in the molecular systems. However, these functions do not clearly indicate the quantitative description, or the extensiveness of the atomic interactions. 

The atomistic interaction information obtained, either by experimental or molecular simulations, is of low data discoverability and not directly accessible by data query or other computational means. For example: What is the reliable mechanism to locate previously published works on some similar interactions that may not involve the same sets of molecules? How can such interactions be distinguished from one another at the atomistic levels, and from such, is it possible to correlate quantitatively their inter-relationships in some given conditions?

To this end, the DL_ANALYSER Notation for Atomic Interactions (DANAI) has been developed to address these issues. DANAI is a standard notation scheme of atomic expression implemented in DL_ANALYSER [13]. DL_ANALYSER is a software program written to carry out post-analysis on system trajectory files produced by the DL_POLY [14] molecular dynamics simulation package.

By making use of the DL_F Notation [15], the standardised, universal notation for atom typing in molecular simulations, DANAI provides easy-to-understand and easy-to-interpret expression syntax to annotate the atomistic interactions in the molecular systems. The DL_F Notation can precisely indicate the actual chemical nature of every atom in molecular systems and is independent of the force field schemes employed in the molecular simulations. Since atoms that are involved in an interaction are expressed in the DL_F Notation, the DANAI expression can be easily interpreted by modellers and experimentalists, as well as computational means. It contains the actual chemical information and annotates a given set of localised atomic interactions that can be accessed by means of data analytics. 

**Figure 2:**
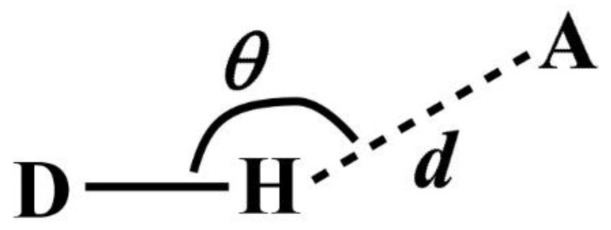
Definition of a hydrogen bond.

Note that DANAI does not contain local geometrical information such as the spatial and orientation arrangement of atoms. Rather, such information is pre-defined as criteria by the users for the software to identify the atomic interactions as described according to the DANAI expression. In other words, an interaction is identified based on a given set of critical values. For instance, in the case of HB, the H-acceptor distance (*d*) and the angular orientation (*θ*) must be specified, as illustrated in Figure 2. DL_ANALYSER will then use these critical values to determine quantitatively the presence of such interactions.

In this paper, the DANAI syntax will first be described. After that, the atomic interactions between ethanoic acid molecules will be used as an example to illustrate the use of the DANAI notation. This was achieved by first constructing a force field model where the atom labels were described in the DL_F Notation using DL_FIELD [15]. After that, the MD simulations of pure ethanoic acid were carried out using the DL_POLY_4 program. Lastly, the DL_ANALYSER program was used to analyse the molecular trajectory produced from the simulations. 

## 2. DANAI Notation Syntax

By following the flavours of the DL_F Notation, DANAI avoids the use of too many cryptic symbols by keeping them to a minimum. This enables users to interpret easily the interaction expressions without regular references to a manual.

In a DANAI system, the atomic interactions can be classified into two types: the macro-interactions and the corresponding sets of micro-interactions. A macro-interaction refers to a specific type of non-bonded molecular interaction between two *Chemical Group*s (*CGs*, as defined in the DL_F Notation) in a general sense. The micro-interactions are various modes of local interactions which consist of a set of atoms belonging to the *CGs* that participate in the interactions as specified by the corresponding macro-interactions. In other words, both the macro-interaction and micro-interaction must be specified in a DANAI expression to provide a complete description for an atomic interaction.

### 2.1. Macro-Interactions 

The macro-interactions are expressed with the general format: *A_CGI1_CGI2*, where *A* is the interaction type and *CGI* is the *Chemical Group Index* which is the unique numerical value for a given *CG* in the DL_F Notation. Some examples of macro-interaction types are shown below:

*DD*: Dipole–dipole interactions, including dipole–induced dipole.

*HB*: Hydrogen bonding, a special case of *DD*.

*ID*: Dispersive (induced-dipole–induced-dipole), van der Waals type of interactions.

*HP*: Hydrophobic interactions (a special case of *ID*, as between alkyl groups).

*EI*: Electrostatic interactions (such as between cations and anions).

*CD*: Charge–dipole interactions (such as between ions and polar atoms).

*PS*: The π- π parallel stacking interactions (such as between two aromatic rings).

*PD*: Parallel displaced π - π stacking interactions (such as between two aromatic rings).

*PT*: T-shaped π - π stacking interactions (such as between two aromatic rings).

*PI*: Ion- π interactions (as between a cation and an aromatic π -delocalisation system).

Some examples of macro-interactions between different *CG*s are illustrated in the Supplementary Materials.

### 2.2. Micro-Interactions 

The interactions are expressed with the general format: *[**S**a] atomic_interaction*, where ***S*** is the general description of the topological structure as a result of the interaction and, *a* is the number of *CGs* involved in the interaction that form such structure. Some examples of ***S*** are shown below.

***J***: A junction or a bifurcation intersection.

***R***: Interactions that form a closed-loop structure, such as a ring.

***L***: Linear structure.

***C***: Complex structure containing a mixture of the above-mentioned structures.

For instance, ***L***3 means a linear micro-interaction that involves three *CGs*. ***R***2 means a micro-interaction that involves two *CGs* that form a ring enclosure. A good example of the ***R***2 interaction would be dimerization of two carboxylic groups via the intermolecular hydrogen bonding. The *atomic_interaction* is a line of text that annotates the atomic species involve in the interaction. These atomic species solely consist of the members belonging to the *CGs* as described in the corresponding macro-interaction.

Apart from the atomic species, the *atomic_interaction* expression can also contain the following symbols:

**:**  Represents the non-bonded interaction, of which the type is described according to the macro-interaction. Every *atomic_interaction* expression must always include at least one non-bonded interaction. This symbol can be located at either side of an element, indicating the direction and the neighbouring atom with which it forms the interaction.
– A chemical (covalent) bond between two atoms.
# The remaining part of the same *CG* that *does or does not* participate in any non-bonded interactions. This is usually used when two interactions occur at different parts of a functional group, with the remainder represented as an “#”. Atoms that are collectively represented by the “#” symbol are covalently bonded and will not be considered as part of the criteria to identify an interaction. In other words, instead of using the “#” symbols, these atoms can be explicitly expressed with the element symbols in the lowercase. For example, for the carboxylic_acid *CG* (COOH), the corresponding DANAI expression oE#h is equivalent to oE-c-o-h.
@ The remaining part of the same *CG* that *does not* participate in the non-bonded interaction, as specified by the macro-interaction. This is different from “#” whereby, atoms that are collectively represented by the “@” symbol will be used as part of the criteria to identify an interaction. In other words, instead of using the “@” symbol, all these atoms can be explicitly expressed with all the element symbols in uppercase. For example, for the carboxylic_acid CG (COOH), the corresponding DANAI expression oE@h is equivalent to oE-C-O-h.
(X) The bracket is used to indicate an atom or a group of atoms X that forms a branch or part of a molecule. For example, consider a general DANAI expression A:B(X)-C. This means atom A forms a non-bonded interaction with atom B, which is covalently bonded to both atom X and atom C. Note that atom X is not bonded to atom C. On the other hand, the DANAI expression A-B(:X)-C means atom A is bonded to B, which in turn is bonded to atom C. The atom X forms a non-bonded interaction with atom B only and is not bonded to any other atom.
< > The arrow bracket is used to indicate a ring enclosure. This is usually used at the first and the last element in an *atomic_interaction* expression. With the exception of atoms in brackets (X), all atoms contained within the < > bracket are considered to be the members that form the ring structure. These brackets are usually used in complex interactions structures, ***C***. They are also used to describe collectively a group of atoms (see below).

### 2.3. DANAI Expression Rules

The following lists special rules when using DANAI.

(1) In general, all atomic species are described in the DL_F Notation. However, the element symbols can be expressed in either uppercase or lowercase, which indicates the extent of the non-bonded interactions on the atom for DL_ANALYSER to identify. If the atom is specified in the uppercase, then only such an atom involved precisely in the number of interactions, as defined in the *atomic_interaction* expression, will be considered. If the atom is specified in the lowercase, then such an atom will always be considered irrespective of the number of different interactions involving this atom. In other words, in addition to the user-defined critical values, the selection criteria for a given set of atoms that are described in an *atomic_interaction* expression is also dependent upon the upper or lower-case of the atomic symbols.

For example, consider the DANAI expression A:B where A and B are some atomic symbols. The expression states that atom A formed a non-bonded interaction with atom B. Since the atoms are expressed in capital letters, this means that DL_ANALYSER will only identify and count such interaction *if and only if* there is a non-bonded interaction between A and B and no other similar interaction exists between atom A or atom B and any other atoms. Conversely, the DANAI expression a:b means that DL_ANALYSER will identify an interaction configuration if at least one such interaction exists between atom A and atom B, irrespective of any other interactions may also exist between atom A or atom B with any other atoms.

(2) For a ring structure, ***R***, the first and the last element symbols in the *atomic_interaction* expression are always referred to by an identical atom, which indicates the extent of the ring enclosure.
(3) For interactions involving delocalised *π*-electrons, the atomic species that participate in the electron delocalisation can be described collectively in the *atomic_interaction* expressions by using the arrow brackets, < >. For example, carbon atoms in aromatic rings are described as <*C6*> and <*C501*>, where the numbers *6* and *501* are the CGI values for benzene and pyridine, respectively.

The section below shows specific examples of using DANAI expressions for carboxylic acid interactions.

### 2.4. Examples

It is known that carboxylic acids form HB interactions at the carboxylic functional groups. The general DANAI expression to describe such interactions is *HB_20_20*. The value 20 in the macro-interaction expression is the *CGI* for the carboxylic_acid *CG*, as defined in the DL_F Notation (see the Supporting Information in Ref. [15]). The corresponding micro-interactions would, therefore, only express various modes of HB interactions involving two or more carboxylic groups.

**Figure 3:**
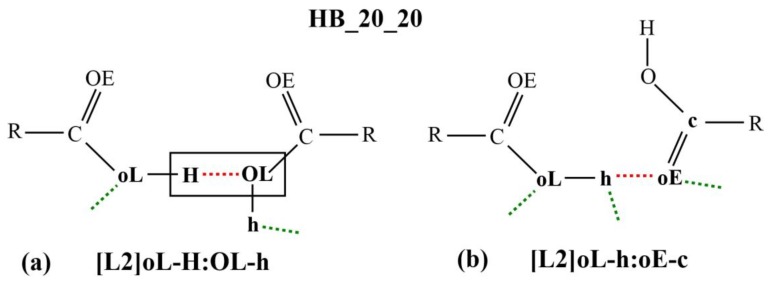
Diagrammatic illustrations of two (micro-interactions) different modes of HB interactions between two carboxylic groups for HB_20_20: (**a**) HB interaction between two hydroxyl groups; and (**b**) HB interaction between a hydroxyl hydrogen atom and a carbonyl oxygen atom. The red dotted lines refer to the HB interactions which are indicated as the symbols **:** in the DANAI expressions. The green dotted lines refer to some other HB interactions with other atoms (not shown).

Figure 3a,b shows two examples of the micro-interactions. Many more examples are shown in the Supplementary Materials. According to the DL_F Notation, the atomic species that are involved in the DANAI micro-interaction expression consist of the carbonyl group, C20 and O20E, and the hydroxyl group, O20L and H20O. Since only the carboxylic groups are involved in the HB interactions, the atomic species can be abbreviated as C, OE, OL and H, respectively; or c, oE, oL and H, respectively, depending on the identification criteria for the number of HB.

The micro-interaction expression in Figure 3a stipulates strict identification criteria on the atoms labelled H and OL (enclosed in a rectangular box) where only one HB interaction is allowed between them. This HB is represented as the red dotted line in the diagram or as the symbol **:** in the corresponding micro-interaction expression, H**:**OL. DL_ANALYSE will not consider an interaction configuration for this micro-interaction if the configuration contains additional HB interactions detected at either the H atom or the OL atom. On the other hand, there is no identification criteria imposed on the atoms that are expressed in the lowercase, oL and h. For this reason, the interaction also can be expressed simply as [L2]H:OL.

Similarly, in Figure 3b, all the atoms that were involved in the interactions are expressed in lowercase. This means that a configuration interaction will be accepted as a count for this interaction expression as long there is at least one HB detected between the oE and the h atoms. For this reason, the interaction expression can be abbreviated simply as [L2]h:oE.

In addition to HB interactions, the non-polar alkyl groups in carboxylic acid molecules participate in dispersive van der Waal interactions, more commonly known as the hydrophobic interactions (HP). The macro-interaction is expressed as *HP_1_1* (interactions between the alkyl groups). For example, consider the ethanoic acid molecules, the HP interactions occur at the primary alkyl carbon (methyl). According to the DL_F Notation, the methyl carbon is C1p, where the value 1 is the unique *CGI* value referring to the alkane *CG*. Some examples of such interactions are shown in the Supplementary Materials.

In the following sections, the use of DANAI is demonstrated, by making references to the analysis of the HB interactions and HP interactions in pure ethanoic acid molecular systems, obtained from MD simulations (See Simulation Methods).

## 3. Results

The criteria to define a HB were set with *d* = 2.5 Å and *θ* = 120° (see Figure 2). This means DL_ANAYSER will identify a HB interaction only if the distance between a hydrogen atom and an acceptor (which is the oxygen atom in this case) is less than or equal to 2.5 Å and the angle where H is the apex is more than or equal to 120°. These values were used as they satisfy the detection of HB in most cases [16]. We have inspected the radial distribution function between the OE and H atoms and found that the first major peak, indicating the HB formation between the two atoms was located well within the distance of 2.5 Å. Additional analysis has also been carried out by adjusting the angle *θ* by ± 10° about the chosen value of 120° and found that there was no significant difference in the results that may otherwise lead to different conclusions than what is discussed below. 

To quantify the HP interactions, the critical distance was set to *d* = 4.5 Å between the alkyl carbon atoms. This means that DL_ANALYSER will determine a HP interaction as significant if the distance between the C1p atoms is less than or equal to *d*. This is the only criterion that is used to determine the interactions and the DANAI expressions for the HP interactions will only consist of the alkyl carbon atoms. Although hydrophobic interactions are known to be long range in nature compared to a typical covalent bond length [17], the value of the critical distance was so chosen such that only “direct contact” with the nearest neighbours among the alkyl carbon atoms would be selected, which ensured that no other atom could be located between two neighbouring alkyl carbon atoms.

### 3.1. HB Interactions in Ethanoic Acid, HB_20_20

Table 1 shows a list of micro-interactions for HB in ethanoic acid molecules. These values are expressed in terms of the average number of micro-interactions identified according to the DANAI expressions. The corresponding diagrammatic representations of these interactions can be found in the Supplementary Materials.

It can be seen that HB formed predominantly between the carbonyl oxygen and the hydroxyl hydrogen (H:OE and h:oE) (Expressions 4–6), and much more so than they formed between the hydroxyl groups (Expression 1–3). Both sets of expressions show the expected trend where the number of interactions increases as the criteria to identify the HB interaction broadened, as indicated by the changes of the element symbols from capital letters to small letters in the DANAI expressions. For example, consider Expression 1 with respect to Expression 3, of all the dimeric HB interactions involving the hydroxyl groups (OL-H:OL-H), only about 1.5% of such interactions occur in isolation, with no other additional hydrogen bond apart from that between the OL and the H. Conversely, for the interactions between the hydroxyl hydrogen and carbonyl oxygen (OL-H:OE-C), about 72% occurred in isolation, with no other additional hydrogen bond apart from that between the OE and H (Expression 4). On the other hand, Expression 10 shows only a small count for a bifurcated HB interaction (double hydrogen bond), involving two polar hydrogen atoms (h) and a carbonyl oxygen (oE).

The results in Table 1 show that the carboxylic groups do not appear to form an extensive branched HB network in the liquid phase. Note that Expression 10 is the only dominant form identified for a double hydrogen bond structure, interacting via the h:oE(c):h. Other forms, such as the [J3]oE:h(oL):oE or the [J3]oL:h(oL):oL interactions have also been identified but with much lower count values.

Expressions 7–9 show various types of linear interactions involving three carboxylic groups. Interestingly, there is a difference between Expressions 7 and 8, indicating the dynamic stability of the HB interaction between the hydroxyl groups may possibly depend on the other HB interactions at the carbonyl oxygen from the same carboxylic groups. Expression 9 indicates a large number of interactions and this shows that most of the interactions identified in Expressions 4–6 may well be the subset that formed part of the greater interaction structures.

To investigate the inter-relationships between these different sets of interactions, the correlation coefficients between any two interactions, *C_x–y_* can be calculated and is defined as

$$C_{x-y}=\;\frac{\langle \Delta C_{x}.\Delta C_{y}\rangle }{\sqrt{\langle \Delta C_{x}^{2}\rangle \langle \Delta C_{y}^{2}\rangle }}$$

$$\Delta C_{i}=\;C_{i}-\;\mu_{i}$$

where, for a given DANAI expression *x* (as shown in Table 1), *C_i_* is the total number of hits for the Expression *x* in an MD instantaneous time frame and *μ_i_* is the corresponding mean over all MD time frames. The quantity is normalised such that Expression *x* and Expression *y* are completely correlated when *C_x–y_* = 1.0 and completely uncorrelated when *C_x–y_* = 0.0. Table 2 shows the values of *C_x–y_* for all possible combination of the above-mentioned interaction Expressions.

It is shown that the overall dimeric interactions OL:H (Expressions 1–3) are anti-correlated to the overall OE:H interactions (Expressions 4–6), which are indicated by large negative *C_x–y_* values. For instance, for *x* = 3 (the overall oL:h), the *C_x–y_* values vary as −0.834, −0.653 and −0.628 when *y* = 4, 5 and 6, respectively. This means that both interaction types are in competition with each other, that one interaction is increased at the expense of the other and *vice versa*. Conversely, the large positive *C_3–y_* values of 0.617 and 0.853 when *y* = 7 and 8, respectively, strongly suggests that most of the overall oL:h interactions form part of the linearised [L3] structures.

When considering the overall oE:h interactions (*x* = 6), it has a large *C*_6*–y*_ value of 0.915 when *y* = 9. This suggests the oE:h interactions tend to form part of the larger linearised H-bond structures involving more than two carboxylic groups. In fact, *C*_4*–*9_ = 0.676 and *C*_5*–*9_ = 0.616 further shows that it is not common to form a branched, hydrogen bond network.

### 3.2. HP Interactions in Ethanoic Acid

Table 3 shows a set of HP expressions along with the average number of interactions (hits) identified. The corresponding diagrammatic representations of these interactions can be found in the Supplementary Materials. The results clearly show that a large portion of methyl carbon atoms interact linearly involving at least three C1p atoms (Expression 3 in Table 3), with a value (~2131) greater than the total number (674) of methyl carbon atoms in the molecular system. This shows the methyl groups formed clusters with multiple hydrophobic contacts. This is shown in Expression 5, of which there are on average about 410 methyl groups that were surrounded with at least three other methyl groups; and Expression 6 shows there are about 178 methyl groups that were surrounded with at least four other methyl groups.

When comparing Expression 1 and Expression 2, only a tiny proportion (0.18%) of methyl groups formed isolated dimeric hydrophobic contacts. Interestingly, Expression 4 indicates there were very few that formed trimeric ring-like structures.

Table 4 shows the correlations coefficients for all possible combinations of the HP interactions shown in Table 3. The large positive values of *C*_5*–y*_ relative to *y* = 2 and 3 strongly indicate that the c1p:c1p and c1p:c1p:c1p interactions were in fact members of some larger hydrophobic clusters. Similar conclusions can also be reached for *C*_6*–y*_.

### 3.3. Correlations between HB_20_20 and HP_1_1 in Ethanoic Acid

To investigate how the HB interactions in ethanoic acid affects the HP interactions and vice versa, the cross-correlation calculations between the interactions were performed using the DL_ANALYSER program. It was found that, for all possible combinations of cross correlations between the HB and HP interactions, the magnitudes of correlation coefficients have values that are less than 0.1. This means the formation of HB structures among the carboxylic groups was independent of the formation of HP structures.

## 4. Discussions

This paper introduces DANAI, a syntax expression system based on the DL_F Notation, to annotate localised atomic interactions between molecules and classify according to the type and nature of the interactions. 

By making references to the MD simulations of pure ethanoic acid it is shown that, by using DANAI, a variety of different localised atomistic structures can be identified to provide a more complete and detailed quantitative picture of the highly complex HB and HP interaction networks in the liquid system. From a set of different DANAI expressions and the corresponding interaction correlation results, it was found that “head-to-tail” is the predominant HB interaction between the carboxylic groups, which involves the carbonyl oxygen (OE) as an acceptor and the polar hydroxyl hydrogen (H). In the case of hydrophobic interactions, the methyl groups tend to form multiple contacts between one another, resulting in a large supercluster extended across the whole molecular system.

The fact that H:OE (head-to-tail) interactions are more common than the hydroxyl (tail-to-tail) interactions, OL:H, can be understood in terms of the vicinity of the polar hydrogen atoms for the latter interactions. Due to the repulsive electrostatic interactions between these hydrogen atoms, the hydrogen bond distances at OL:H tend to be longer and hence weaker compared with those of H:OE, even though the hydroxyl O-H is more polar than the carbonyl C=O group.

**Figure 4:**
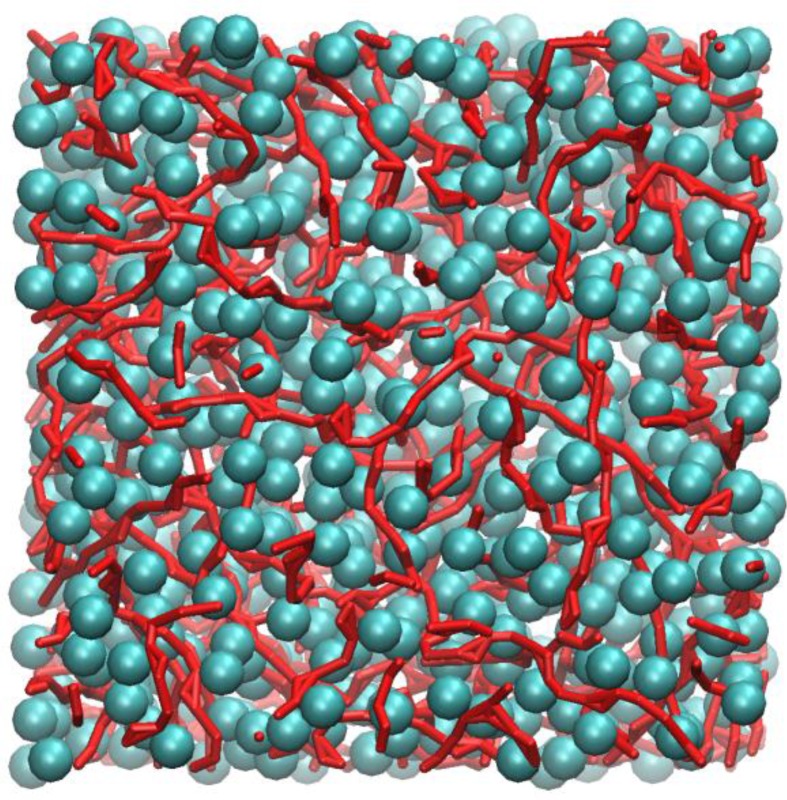
MD snapshot of ethanoic acid liquid at time = 4.44 ns, with depth fading effect.

Figure 4 shows an instantaneous snapshot of the molecular configuration at time = 4.44 ns. All methyl carbon atoms that are in nearest contact with at least one other neighbour are shown as cyan spheres. The carboxylic groups that participate in HB are represented as red streaks. For clarity purposes, the alkyl hydrogen atoms are not shown. In this particular snapshot, there are four methyl carbon atoms that did not participate in close HP contact with any other methyl groups and three carboxylic groups that did not participate in HB interactions. These are not shown in Figure 4.

Inspection of Figure 4 shows that the methyl carbons formed a “sponge-like” superstructure and distributed evenly over the whole molecular system. By tracing out all closest neighbour contacts of every methyl carbon, it was found that the supercluster consists of 664 methyl carbons, which is almost the entire number of methyl carbon atoms (674) contained in the system model. Three isolated dimers were also identified, probably temporarily detached from the supercluster due to thermal fluctuations. 

Figure 4 also shows that most carboxylic groups participated in HB, forming individual linearized macro-structures, as traced out by a series of red streaks, intertwining with pockets of hydrophobic regions and seldom overlapped with one another. The length of these linear chains can consist of anywhere from two to ten carboxylic groups, the majority of which interact via the “head-to-tail” mechanism of the carboxylic acid groups (OE:H). Occasionally, branching also occurred, mostly due to the bifurcated structures, involving two polar hydrogen atoms interacting with the carbonyl oxygen atoms, h:oE:h (Expression 10 in Table 1). Visual inspection of the molecular configuration apparently showed that, while the end-to-end distance of the longest chain appeared to be smaller than the size of the simulation box, the corresponding contour length of the chain may not. This poses an important question, i.e. whether larger box size is needed to effectively model these large structures, and this certainly warrants further investigations.

Interestingly, the cross-correlation analysis shows that the formation and evolution of the HB and HP interaction structures occurred independently from each other. These chains of HB structure are thought to be the reason for the unusual physicochemical behaviour whereby ethanoic acid has unusually high boiling and melting points compared with other comparable organic molecules such as ethanol. Furthermore, the extensiveness of the HP interactions in ethanoic acid indicates the importance of alkyl chains in contributing to high boiling and melting points when the alkyl chain length of carboxylic acids is increased [18].

There are some previous studies [11,19,20,21] on the dimerization of carboxylic acid involving two carboxylic groups, acting both as the hydrogen-bond donors and acceptors. However, in our model, such an interaction configuration ([R2]c-oL-h:oE#h:oE-c) only occurred with a very low count value (<1) and is therefore not shown in the results tables. Previous works show that such a configuration is usually not common in aqueous solutions but is more important in the gas phase and in non-polar solvents where hydrophobic interactions are important. Our work is also in agreement with such observations since the ethanoic acid itself is a polar solvent.

Without resolving to the use of some arbitrary diagrammatic and pictorial illustrations, this paper demonstrates the use of DANAI to extract atomistic information from molecular simulations for data analytics purposes. DANAI is especially useful for annotation of localised atomic interactions involving members of atoms from different functional groups. The actual number of an interaction identified depends on some critical values set for the identifications. Even then, useful conclusions can be derived provided the criteria set is in some “sensible range” of values. Note that since DANAI expressions contain no information about the overall geometrical orientation of the interacting atoms, some local order parameter calculations will still need to be carried out if one were to quantify certain specific geometrical structures.

DANAI is thought to be useful in the analysis of atomistic simulation models, especially those of organic molecules. For instance, organic crystals, solvent–solute interactions, and the solubilisation and crystallisation behaviour of organic molecules. In addition, DANAI can potentially be useful in cheminformatics on predictions and statistical model constructions for the atomic interactions of molecular systems.

Note that DANAI contains an expression concept that is not necessarily specific to a software package or the notation of atomic symbols. However, this paper demonstrates that, by making use of the DL_F universal atom typing for the atomic symbols in the MD simulations and in the DANAI expressions, the trajectory data extracted from the molecular simulations can be analysed as is, without further data transformation, producing results that contain the actual chemical information that is independent of the force field schemes used for the molecular models.

DANAI was first implemented in DL_ANALYSER version 1.4. The current version 2.0 is able to analyse a set of interactions for the alkane, carboxylic_acid and alcohol *CGs*. Future development of DANAI in DL_ANALYSER will include more different types of molecular interactions, such as interactions involving the delocalised π-electrons for aromatic systems.

## 5. Simulation Methods

A molecular system of simulation box size 40 Å × 40 Å × 40 Å that consists of pure ethanoic acid with density 1.05 g/cm^3^ was constructed using DL_FIELD version 4.1.1 program [22]. This corresponds to 674 ethanoic acid molecules contained in the system model. The option in DL_FIELD was selected to produce the DL_POLY force field files (the CONFIG and FIELD files) where the atom labels were expressed in the DL_F Notation. The MD simulations were then carried out using DL_POLY [14] version 4.07. All analysis of the results was carried out using DL_ANALYSER [13]. The graphical output of Figure 4 was generated using VMD [23].

The OPLS2005 force field [24,25] was used to model the molecular system. The van der Waals and coulombic real space cut off were set to 9.0 Å. The coulombic interactions were treated by means of SPME [26]. The SHAKE algorithm was used to constrain the hydrogen-containing bonds and the tolerance limit was set to 10^−4^ Å.

Initially, the system was equilibrated in the NVE ensemble by heating the system from 10 K to 300 K over 1 ns with the temperature scaling applied at every time step. After that, NVT ensemble was used and the simulation run for another 300 ps at 300 K. It was then changed to NPT ensemble at 1 atmospheric pressure and the equilibration was carried out for a further 500 ps, before the sampling runs. All temperatures and pressures were maintained using the Langevin formalism [27] with the coupling constants set to 0.4 ps and 1.0 ps, respectively. During the sampling process, a fixed time step of 2 fs was used to update the atomic trajectories and the atomic configurations were written to the trajectory file every 2000 steps (4 ps) for a total of 7 ns. This result in a total of 1750 trajectory frames being produced. Post analysis was carried out using DL_ANALYSER on each frame and the total number of interaction identified, or counts, for a DANAI expression was recorded. The average number of interactions, *μ_i_*, for a DANAI expression *i* was obtained by summing up all the number of interactions for the expression *i* recorded for each frame and averaged over all the trajectory frames. The fluctuations, as shown in Table 1 and Table 2, are the standard deviation of *μ_i._* To check for the consistency of the sampling averages, the simulations were repeated using different starting velocities to produce a completely different set of trajectory frames. Subsequent analysis on these frames shew that the *μ_i_* values calculated were similar within the statistical limits when compared with those obtained from another set of trajectory frames. In addition, the simulations were also repeated using another force field scheme, namely, the CVFF (consistent valence force field) [28]. It was found that, although some of the results generated between the OPLS2005 and CVFF are different within the statistical limits, the overall qualitative trends in the average hits and correlation results remain consistent and in agreement between the force field schemes. However, the OPLS2005 is chosen as the basis of the investigation in this paper since it is a newer force field compared with CVFF. The latter was fitted to handle a wide range of small organic molecules, whereas OPLS2005 was fitted over a much wider range of organic molecules including drug molecules for the condensed phase simulations. The OPLS2005 force field is the enhanced variant of the original OPLS-AA [24] that has not only retained most of the parameters from the original force field but also broaden the coverage of pharmaceutically relevant organic molecules by including new atom types and parameters [25]. In fact, previous works had used the OPLS-AA parameters on the investigation of the dimerization of carboxylic acids and compared favourably with those obtained from the quantum-mechanical calculations [29].

Note that, in addition to the DL_POLY’s HISTORY files, DL_ANALYSER can also recognise other file formats such as the PDB and *xyz* files, of which the trajectory files produced from most other MD packages can readily be converted to these formats. DL_FIELD, DL_POLY and DL_ANALYSER are three independent pieces of software that can be used as an integrated software infrastructure for carrying out molecular simulations, from force field model preparation to the simulation run and results analysis. These programs are available to individuals under an academic license, which is free to academics pursuing scientific research of a non-commercial nature. Daresbury Laboratory is the sole centre for distribution of the software. To obtain a copy of the software, please visit http://www.ccp5.ac.uk/software.

## Figures and Tables

**Table 1:** A selection of DANAI expressions for HB interactions identified by DL_ANALYSER between the carboxylic groups in ethanoic acid (HB_20_20).

DANAI Expression, *i*	Average Number of Interactions, *μ_i_*
1. [L2]OL-H:OL-H	1.69 ± 1.27
2. [L2]oL-H:OL-h	79.34 ± 7.58
3. [L2]oL-h:oL-h	113.50 ± 8.75
4. [L2]OL-H:OE-C	418.90 ± 14.65
5. [L2]oL-H:OE-c	504.61 ± 10.76
6. [L2]oL-h:oE-c	581.28 ± 8.50
7. [L3]oL-h:oE#oL(h):h-oL	59.64 ± 6.17
8. [L3]oL-h:oE#h:oL-h	92.95 ± 7.38
9. [L3]oL-h:oE#h:oE-c	477.39 ± 13.60
10. [J3]oL-h:oE(c):h-oL	22.32 ± 4.35

**Table 2:** Correlation coefficients, *C_x–y_*, for HB_20_20. The bold numbers as shown on the top row and left columns refer to the Expressions *x* and *y* as shown in Table 1.

	1	2	3	4	5	6	7	8	9	10
**1**	1.000	0.056	−0.001	−0.004	−0.080	−0.182	0.005	−0.009	−0.163	−0.063
**2**		1.000	0.764	−0.610	−0.444	−0.811	0.627	0.644	−0.794	−0.253
**3**			1.000	−0.834	−0.653	−0.628	0.617	0.853	−0.651	−0.067
**4**				1.000	0.906	0.526	−0.445	−0.696	0.676	−0.312
**5**					1.000	0.405	−0.286	−0.506	0.616	−0.546
**6**						1.000	−0.482	−0.461	0.915	0.364
**7**							1.000	0.521	−0.457	−0.243
**8**								1.000	−0.562	−0.094
**9**									1.000	0.077
**10**										1.000

**Table 3:** A selection of HP interactions identified by DL_ANALYSER among the methyl groups in ethanoic acid (HP_1_1).

DANAI Expression, *i*	Average Number of Interactions, *μ_i_*
1. [L2]C1p:C1p	1.67 ± 1.28
2. [L2]c1p:c1p	949.36 ± 18.87
3. [L3]c1p:c1p:c1p	2131.01 ± 93.23
4. [R3]c1p:c1p:c1p:c1p	14.22 ± 6.30
5. [J4]c1p:c1p(:c1p):c1p	409.83 ± 14.98
6. [J5]c1p:(c1p:)c1p(:c1p):c1p	177.58 ± 13.70

**Table 4:** Correlation coefficients, *C_x–y_*, for HP_1_1. The bold numbers as shown on the top row and left columns refer to the Expressions *x* and *y* as shown in Table 3.

	1	2	3	4	5	6
**1**	1.000	−0.203	−0.162	−0.029	−0.177	−0.105
**2**		1.000	0.966	0.135	0.869	0.848
**3**			1.000	0.075	0.792	0.891
**4**				1.000	0.118	0.123
**5**					1.000	0.627
**6**						1.000

## Supplementary Materials

Supplementary Materials available: Example uses DANAI with diagrammatic illustrations for various types of molecular interactions. Diagrams illustration of HP interactions for the methyl carbon atoms, together with the corresponding DANAI notation. The HB interactions at the carboxylic groups, together with the corresponding DANAI notation (PDF).
