# Supplementary material for: DL_ANALYSER Notation for Atomic Interactions (DANAI): A Natural Annotation System for Molecular Interactions, Using Ethanoic Acid Liquid as a Test Case
Source: Molecules. 2017 Dec 24;23(1):36. doi: 10.3390/molecules23010036 (PMC5943928; doi:10.3390/molecules23010036)
Supplement: Supplementary file 1 [file molecules-23-00036-s001.pdf]

Supporting Information

DL_ANALYSER Notation for Atomic Interactions (DANAI) – a natural annotation system for molecular interactions, using ethanoic acid liquid as a test case.

Chin W. Yong[1,2] and Ilian T. Todorov[1]

[1]Scientific Computing Department, Science and Technology Facilities Council, Daresbury Laboratory, Sci-Tech Daresbury, Warrington WA4 4AD, UK

[2]Manchester Pharmacy School, Faculty of Medical and Human Sciences, Manchester Academic Health Science Centre, the University of Manchester, Manchester M13 9NT, UK.

Email: chin.yong@stfc.ac.uk

Example illustrations of various types of non-bonded molecular interactions (red dotted lines) using DANAI expressions. The *Chemical Groups* with the corresponding CGI values are as follows (the most complete list is available in the latest version of DL_FIELD program):

1 (alkane), 6 (benzene), 15 (alcohol), 47 (ammonium), 962 (cation), 963 (anion)

HP_1_1

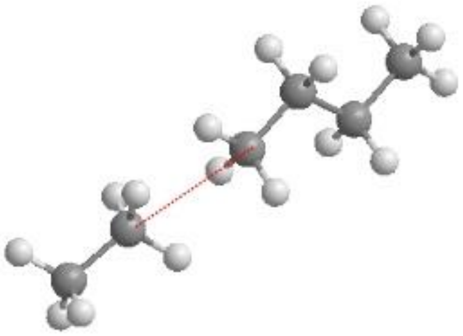

[L2]C1p:C1p

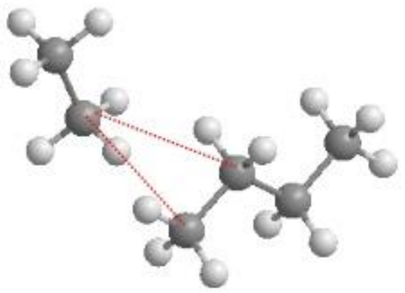

[R2]C1p:C1p-C1s:C1p

PI_6_962

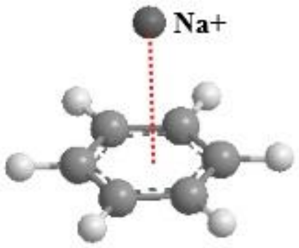

[L2]<C6>:Na962+

PI_6_47

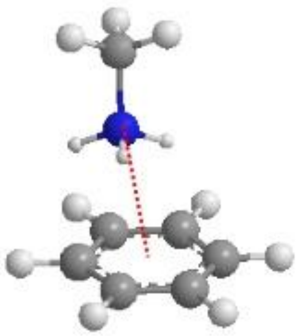

[L2]<C6>:N47+

EI_47_963

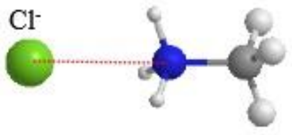

[L2]N47+:Cl963-

CD_15_963

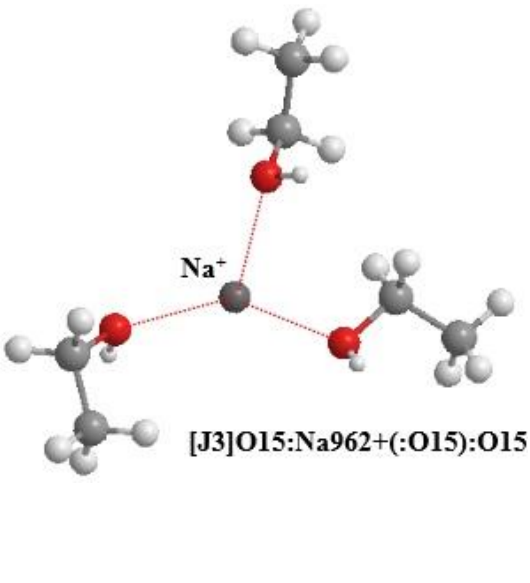

[J3]O15:Na962+(:O15):O15

Macro interaction HP_1_1

The following page shows some examples of the micro-interactions of hydrophobic interactions (HP) between alkyl groups, in diagrammatic illustrations and in the corresponding DANAI notation. For clarity purposes, a sphere collectively represents both the alkyl carbon atom and the alkyl hydrogen atoms as a unified unit. The red dotted lines indicate the HP interactions between the spheres. The value 1 is the unique *CGI* for the alkane *Chemical Group* (*CG*). The letters p, s and t refer to the primary (grey sphere, CH_3), secondary (red sphere, CH2) and tertiary atoms (green sphere, CH), respectively, according to the DL_F Notation.

Some of these micro-interactions were discussed in the paper, as shown in Table 3 of the paper. Note that some of the diagrams are labelled with numbers which correspond to the Expression *i* in Table 3.

Strict criteria are imposed on atoms located within the square boxes where there can be no other HP interaction apart from what is indicated in the DANAI expression, shown as red dotted lines. The element symbols of these atoms are expressed in uppercase letters (C). Atoms that are located outside these enclosures can have any numbers of HP interactions. These atoms are expressed in the lowercase letters (c).

HP_1_1

1

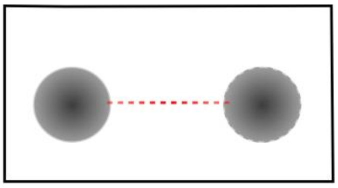

[L2]C1p:C1p

2

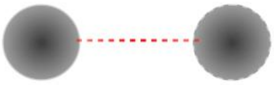

[L2]c1p:c1p

3

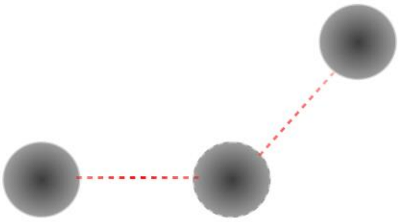

[L3]c1p:c1p:c1p

4

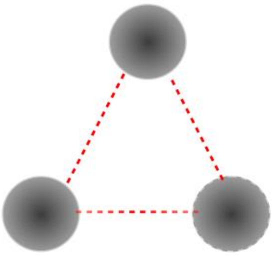

[R3]c1p:c1p:1p:c1p

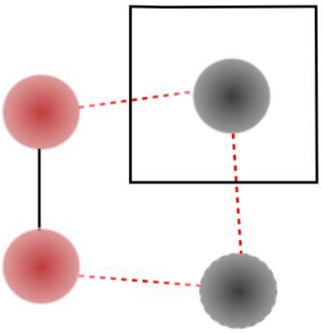

[R3]c1p:c1s– c1s:C1p:c1p

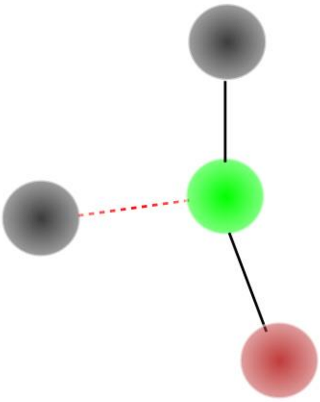

[L2]c1p–c1t(:c1p)–c1s

5

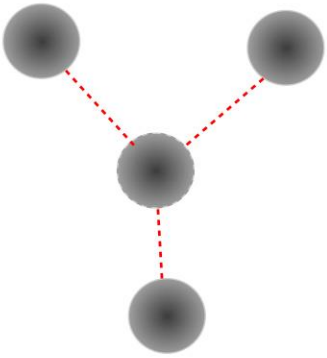

[J4]c1p:c1p(:c1p):c1p

6

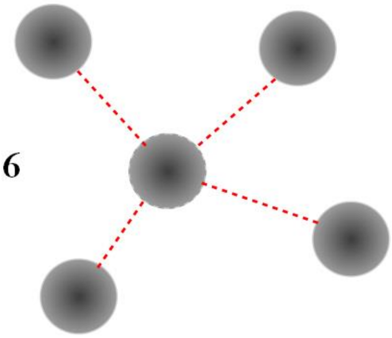

[J5]c1p:(c1p:)c1p(:c1p):c1p

Macro interaction HB_20_20

The following four pages show some examples of the micro-interactions of hydrogen bond (HB) interactions between carboxylic groups, in diagrammatic illustrations and in the corresponding DANAI notation. In some cases, the DANAI expressions can be abbreviated and these are shown in brackets. Some of these micro-interactions were discussed in the paper, as shown in Table 1 of the paper. Note that some of the diagrams are labelled with numbers which correspond to the Expression *i* in Table 1.

The value 20 is the unique *CGI* for the carboxylic_acid *CG*.

Strict criteria are imposed on atoms located within the square boxes and polygons where there can be no other HB interaction apart from what is indicated in the DANAI expression, as highlighted in red dotted lines. The element symbols of these atoms are expressed in uppercase letters. Atoms that are located outside these enclosures can have any numbers of HB interactions. These atoms are expressed in the lowercase letters.

The OE and OL (or oE and oL) refers to the carbonyl oxygen and hydroxyl oxygen atoms, respectively, according to the DL_F Notation. Since only one type of the *Chemical Group* is involved in the interaction, the *CGI* value of 20 has been left out in the DANAI expression. For instance, the interaction [L2]O20L-H:O20E-C20 can be equivalently expressed as [L2]OL-H:OE-C.

HB_20_20, Page 1

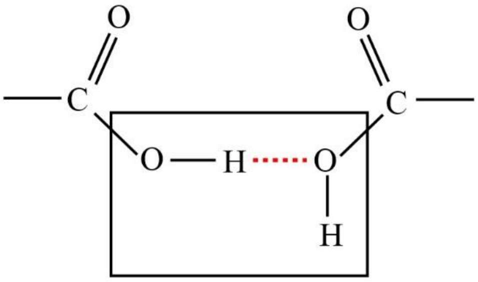

1 [L2]OL-H:OL-H

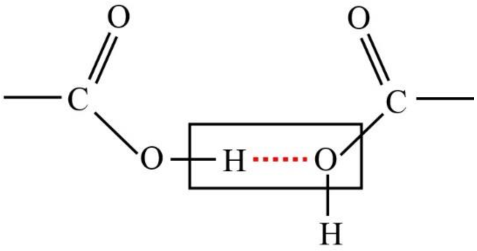

2 [L2]oL-H:OL-h
([L2]H:OL)

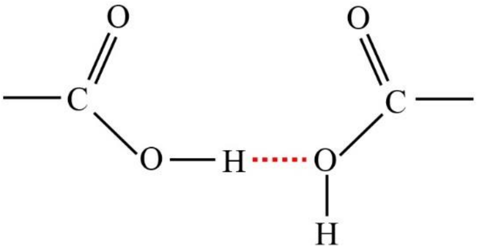

3 [L2]oL-h:oL-h
([L2]h:oL)

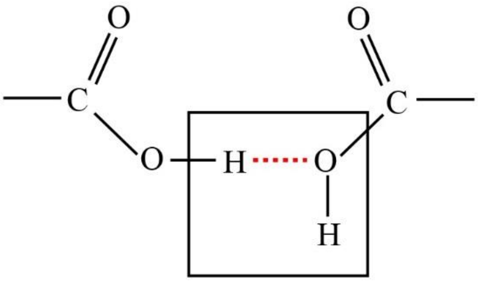

[L2]oL-H:OL-H
([L2]H:OL-H)

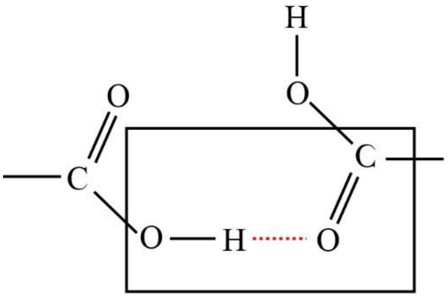

4 [L2]OL-H:OE-C

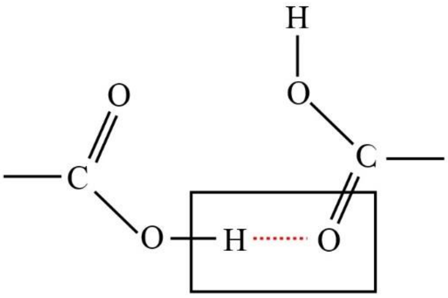

5 [L2]oL-H:OE-c
([L2]H:OE)

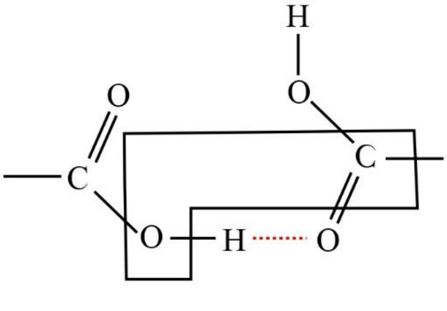

[L2]OL-h:oE-C

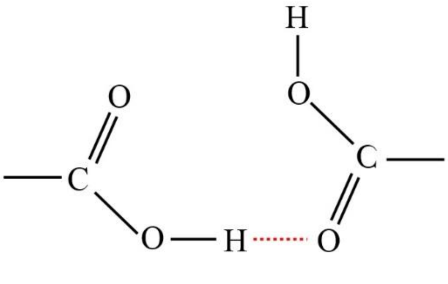

6 [L2]oL-h:oE-c
([L2]h:oE)

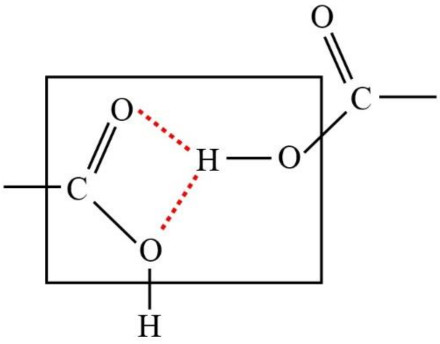

[R2]C-OE:H(OL):OL-C

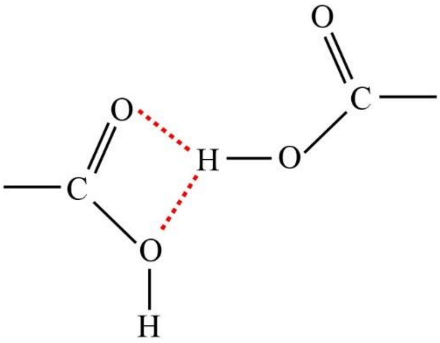

[R2]c-oE:h(oL):oL-c

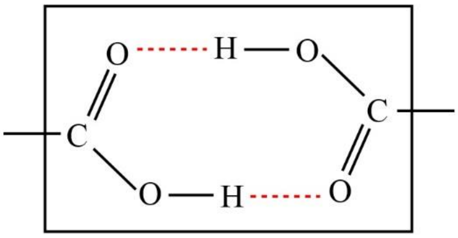

[R2]C-OL-H:OE@H:OE-C

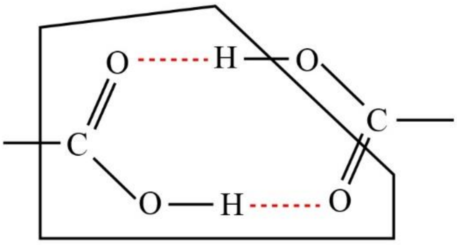

[R2]C-OL-H:OE#H:OE-C

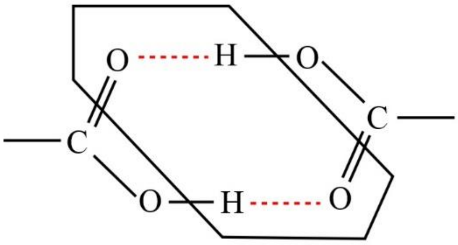

[R2]c-oL-H:OE#H:OE-c

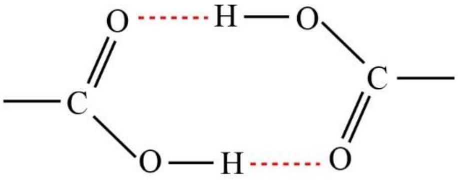

[R2]c-oL-h:oE#h:oE-c

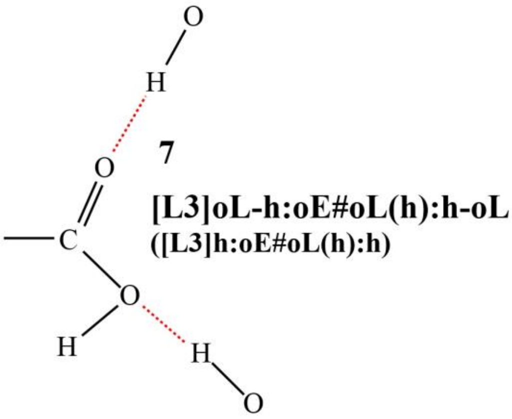

7
[L3]oL-h:oE#oL(h):h-oL
([L3]h:oE#oL(h):h)

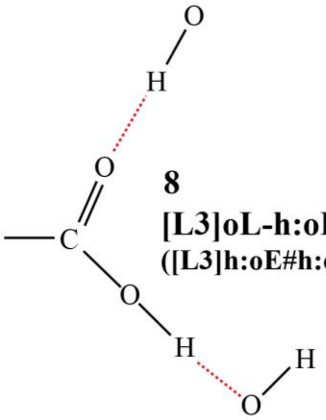

8
[L3]oL-h:oE#h:oL-h
([L3]h:oE#h:oL)

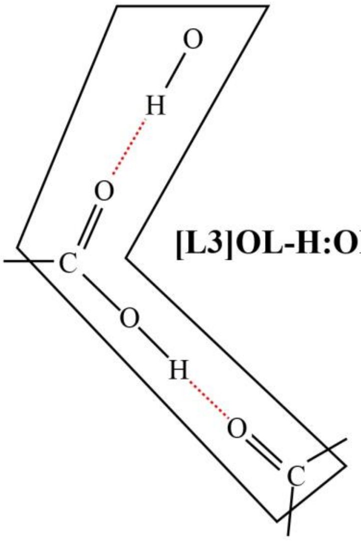

[L3]OL-H:OE@H:OE-C

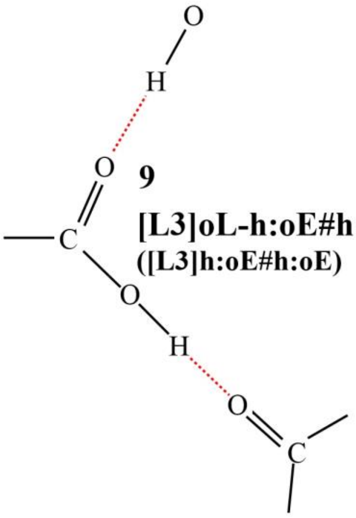

9
[L3]oL-h:oE#h:oE-c
([L3]h:oE#h:oE)

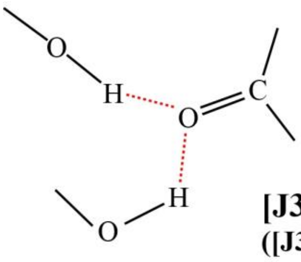

10
[J3]oL-h:oE(c):h-oL
([J3]h:oE(c):h)

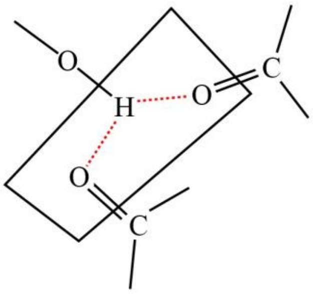

[J3]c-OE:H(oL):OE-c
([J3]OE:H(oL):OE)

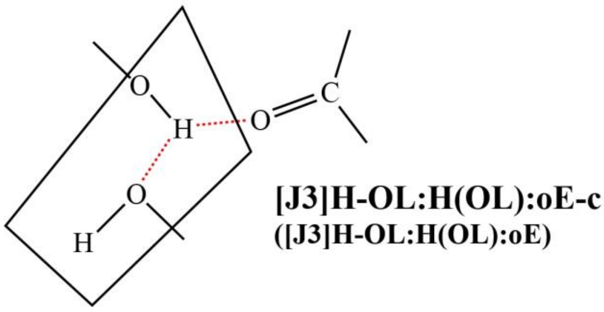

[J3]H-OL:H(OL):oE-c
([J3]H-OL:H(OL):oE)

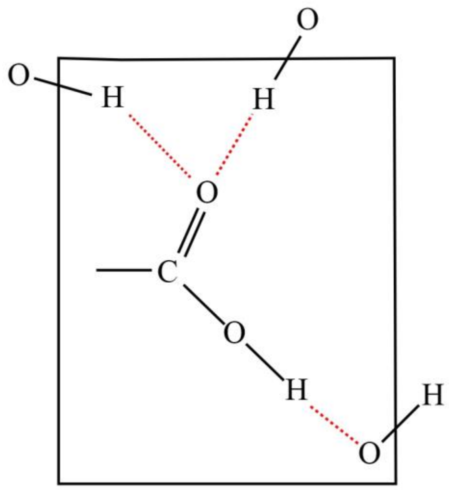

[C4]oL-H:OE(@H:OL-h):H-oL

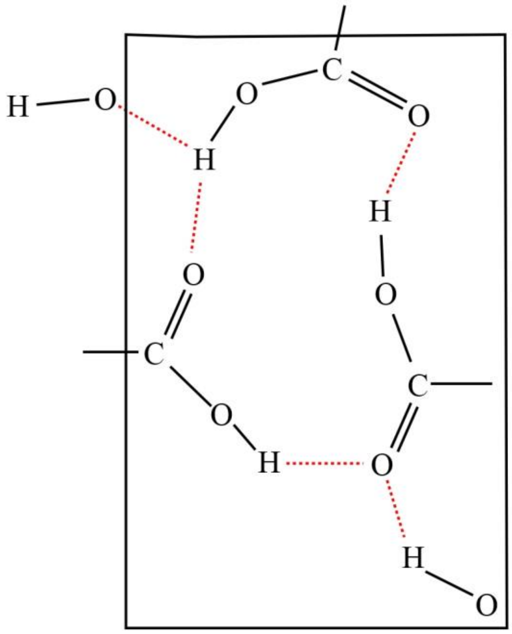

[C5]<C-OL-H:OE(:H-OL)@H:OE@H(:oL-h):OE-C>
